# Patellar Dislocation Patients Had Lower Bone Mineral Density and Hounsfield Unit Values in the Knee Joint Compared to Patients with Anterior Cruciate Ligament Ruptures: A Focus on Cortical Bone in the Tibia

**DOI:** 10.3390/bioengineering12020165

**Published:** 2025-02-08

**Authors:** Yue Wu, Yiting Wang, Haijun Wang, Shaowei Jia, Yingfang Ao, Xi Gong, Zhenlong Liu

**Affiliations:** 1 Department of Sports Medicine, Peking University Third Hospital, Institute of Sports Medicine of Peking University, Beijing 100191, China; wuyue6063@163.com (Y.W.); 2110122708@stu.pku.edu.cn (Y.W.); bysywang@163.com (H.W.); shaoweijia@buaa.edu.cn (S.J.); aoyingfang@163.com (Y.A.); 2 Beijing Key Laboratory of Sports Injuries, Beijing Municipal Science and Technology Commission, Beijing 100191, China; 3 Engineering Research Center of Sports Trauma Treatment Technology and Devices, Ministry of Education, Beijing 100191, China; 4 Tianjin Key Laboratory of Exercise Physiology and Sports Medicine, Institute of Sport, Exercise & Health, Tianjin University of Sport, Tianjin 300381, China

**Keywords:** anterior cruciate ligament, patellar dislocation, bone mineral density, dual-energy X-ray absorptiometry, computed tomography

## Abstract

Anterior cruciate ligament (ACL) rupture and patellar dislocation (PD) are common knee injuries. Dual-energy X-ray absorptiometry (DXA) and computed tomography (CT) are widely used clinical diagnostic tools. The aim was to investigate the characteristics of knee bone mineral density (BMD) in patients with ACL rupture and PD and to explore the relationship between BMD and Hounsfield unit (HU) values. This prospective cross-sectional study included 32 ACL rupture and 32 PD patients assessed via DXA and CT. BMD and CT measurements were taken from regions of interest in the femoral and tibial condyles. Statistical analyses included t-tests and mixed-effects models. The results showed that BMD in the PD group was significantly lower than in the ACL group (*p* < 0.05). The HU values of cortical bone in the femur and tibia differed significantly between the ACL group and the PD group (*p* < 0.05). The BMD of the femur and tibia showed significant correlations with the HU values of cancellous bone and cortical bone (*p* < 0.05). The conclusion was that PD patients had lower BMD and HU values in the femur and tibia compared to patients with ACL ruptures, particularly in the cortical bone of the tibia, and there was a strong correlation between HU value and BMD.

## 1. Introduction

Anterior cruciate ligament (ACL) rupture and patellar dislocation (PD) are common knee injuries in the field of sports medicine, particularly among young and active individuals [1,2]. The annual incidence of ACL rupture is approximately five cases per 10,000 people in the general population [3]. In contrast, the incidence of PD is 5.8 per 100,000 individuals, with a higher rate of 29 per 100,000 among young adults [4]. Following ACL rupture and PD, patients often experience symptoms such as knee swelling, limited mobility, and joint instability, which can affect daily activities, physical function, and quality of life, especially for athletes [5,6,7]. As a result, these conditions are frequently associated with misdiagnoses or missed diagnoses in clinical practice.

Although ACL reconstruction (ACLR) is commonly performed after ACL rupture, several studies have reported a decrease in local bone mineral density (BMD) around the knee joint [8,9]. However, the impact of PD on knee joint BMD remains underexplored. Surgical treatment for both ACL rupture and PD is the current standard approach for addressing knee joint instability [10,11]. These procedures both involve the process of drilling bone tunnels, and both face challenges related to ligament-to-bone healing postoperatively. Minimizing the rate of bone tunnel enlargement and promoting tendon–bone healing are closely linked to the local BMD.

Dual-energy X-ray absorptiometry (DXA) is the conventional method for measuring BMD, but it cannot differentiate between cancellous and cortical bone [12,13]. Computed tomography (CT) is a widely used clinical diagnostic tool that can distinguish between trabecular and cortical bone [14]. However, routine CT imaging only reflects signal strength through Hounsfield unit (HU) values and does not directly quantify BMD [15].

The aim of this study is to investigate the characteristics of BMD changes in the knee joints of patients with ACL rupture and PD and to explore the relationship between BMD and HU values obtained from CT. This research can provide a theoretical foundation for improving the clinical treatment and rehabilitation of ACL rupture and PD.

## 2. Methods

### 2.1. Study Design

This study is a prospective cross-sectional study with 1:1 ACL rupture patients and PD patients. The Ethics Committee of Peking University Third Hospital approved the study (202303303).

This study was registered at www.clinicaltrials.gov (NCT05924178). IRB approval was granted for this study. Informed consent was obtained from all participants prior to enrollment.

### 2.2. Participant Involvement

Volunteers were not directly involved in the design of the study but actively participated in the study. A recruitment advertisement was posted in the outpatient department to attract individuals with ACL rupture and PD patients.

### 2.3. Participants

Participants with unilateral ACL rupture (ACL group) and age-matched patellar dislocation (PD group) were included in this study.

Inclusion criteria for the ACL group were as follows [16,17,18,19]: (1) Patients were ages 18 to 45 years with a first-time unilateral ACL rupture diagnosed via MRI. (2) Time since injury was greater than 4 weeks. The affected knee joint had passed the acute phase, with no significant swelling, pain, inflammation, or limited joint movement, and had basically regained normal range of motion. (3) There were no concurrent injuries to the Posterior Cruciate Ligament (PCL), Lateral Collateral Ligament (LCL), or Medial Collateral Ligament (MCL).

Inclusion criteria for the PD group were as follows [20,21,22]: (1) physiologic trochlea and Dejour type A or B trochlear dysplasia, (2) patellar dislocations (≥2 times), and (3) MRI evidence of medial patellofemoral ligament (MPFL) lesions.

Data collection was conducted between July 2023 and August 2024.

### 2.4. Sample Size

Preliminary experimental results indicated that the BMD at the femoral and tibial ends of the knee joint were selected as the primary indicators for observation. A statistically significant difference was considered if either of these two indicators showed variation. The Least Significant Difference (LSD) method was applied to correct for Type I error, denoted by α. Based on the preliminary experimental results, the BMD at the femoral end of the knee joint in patients with ACL rupture was 1.31 ± 0.28 g/cm^2^, compared to 1.61 ± 0.31 g/cm^2^ in the contralateral knee. The sample size estimation formula for two independent groups was set at α = 0.05 and β = 0.2, yielding a required sample size of 16 participants per group. Based on the BMD at the tibial end of the knee joint (injured side: 0.94 ± 0.14 g/cm^2^, contralateral side: 1.09 ± 0.15 g/cm^2^), the calculations determined that a sample size of 19 cases per group was needed. Taking the larger of these two results, at least 19 patellar dislocation individuals were selected.

### 2.5. DXA Scan Protocol

BMD was measured using a Lunar iDXA (GE Healthcare, Milwaukee, WI, USA) [23,24]. Lumbar BMD was first assessed to rule out primary osteoporosis, followed by measurement of BMD at the injured knee. All standard clinical scans, including lumbar BMD and knee assessments, were performed and analyzed according to the manufacturer’s guidelines [25].

**Figure 1:**
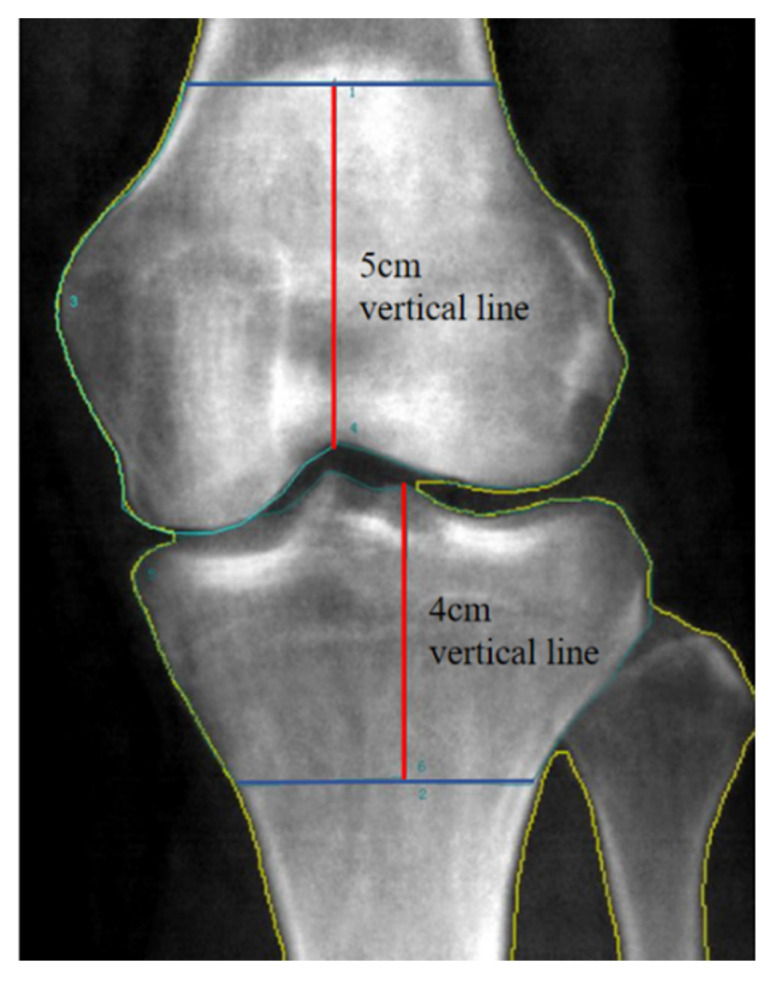
Custom regions of knee BMD interest. Abbreviations: BMD, bone mineral density; ROIs, regions of interest.

For knee BMD measurement, regions of interest (ROIs) were manually outlined. A 5 cm vertical line was drawn from the intercondylar pit of the femur, and a 4 cm vertical line was drawn from the lateral intercondylar ridge of the tibia. This allowed the knee joint to be divided into 4 ROIs: the medial femoral condyle (MCF), the lateral femoral condyle (LCF), the medial tibial condyle (MCT), and the lateral tibial condyle (LCT) (Figure 1).

### 2.6. CT Scan Protocol

#### 2.6.1. Measurement of Femoral HU Values

We selected the layer where the lateral femoral condyle was the most prominent in sagittal plane.

**Figure 2:**
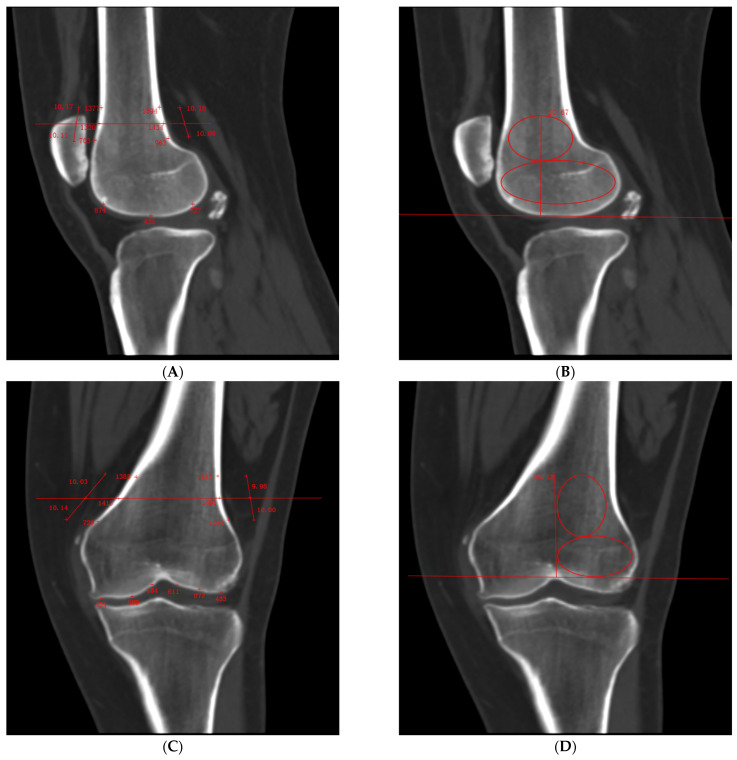

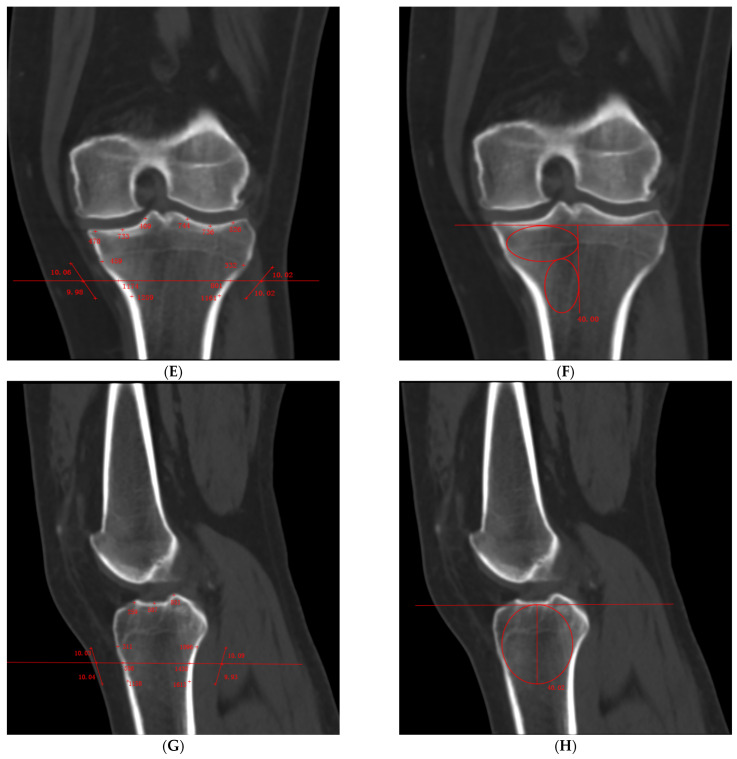
CT scan protocol. Abbreviations: HU, Hounsfield units; SP, sagittal plane; CP, coronal plane. (**A**) Cortical bone HU values in the SP of the femur, (**B**) lateral condyle cancellous bone HU values in the SP of the femur, (**C**) cortical bone HU values in the CP of the femur, (**D**) lateral condyle cancellous bone HU values in the CP of the femur, (**E**) cortical bone HU values in the CP of the tibia, (**F**) medial condyle cancellous bone HU values in the CP of the tibia, (**G**) cortical bone HU values in the SP of the tibia, (**H**) medial condyle cancellous bone HU values in the SP of the tibia.

(1) Cortical Bone HU Value: We measured at the transitional area between the posterior femoral condyle and the shaft (within ± 1 cm above and below this region), taking three points along the anterior cortex of the femur parallel to the posterior femoral surface, as well as three evenly spaced points on the articular surface of the lateral femoral condyle (Figure 2A).
(2) Lateral Condyle Cancellous Bone HU Value: We drew a 5 cm vertical line upward from the highest point of the intercondylar fossa, avoiding the cortical bone, and measured within the fixed region (Figure 2B).

We identified the coronal plane that corresponded to the sagittal plane of the femur.

(1) Cortical Bone HU Value: We measured at the transitional area between the lateral femoral condyle and the shaft (within ± 1 cm above and below this region), taking three points along the medial cortex of the femur parallel to the lateral femoral surface as well as three evenly spaced points on the articular surfaces of both the medial and lateral femoral condyles (Figure 2C).
(2) Lateral Condyle Cancellous Bone HU Value: We drew a 5 cm vertical line upward from the highest point of the intercondylar fossa, avoiding the cortical bone, and measured within the fixed region (Figure 2D).

#### 2.6.2. Measurement of Tibial HU Values

We selected the layer where the tibial intercondylar eminence was the most clearly visible in coronal plane.

(1) Cortical Bone HU Value: We measured at the transitional area between the medial tibial condyle and the shaft (within ± 1 cm above and below this region), taking three points along the lateral tibial cortex parallel to the medial tibial surface as well as three evenly spaced points on both the medial and lateral tibial plateaus (Figure 2E).
(2) Medial Condyle Cancellous Bone HU Value: We drew a 4 cm vertical line downward from the lateral intercondylar eminence, avoiding the cortical bone, and measured within the fixed region (Figure 2F).

We identified the sagittal plane that corresponded to the coronal plane of the tibia.

(1) Cortical Bone HU Value: We measured at the transitional area between the anterior tibial condyle and the shaft (within ± 1 cm above and below this region), taking three points along the posterior cortex of the tibia parallel to the anterior tibial surface as well as three evenly spaced points on the tibial plateau (Figure 2G).
(2) Medial Condyle Cancellous Bone HU Value: We drew a 4 cm vertical line downward from the tibial plateau, avoiding the cortical bone, and measured within the fixed region (Figure 2H).

For each selected layer of the femur and tibia in both the coronal and sagittal planes, we measured the current layer as well as two adjacent layers on either side, collecting data from a total of five layers. We calculated the average value across these five layers for analysis.

### 2.7. Statistical Analysis

Statistical analyses were performed using SPSS 21.0 and R 4.3.1. For normally distributed variables, data were expressed as mean ± standard deviation (x ± SD) and analyzed using the *t*-test and mixed-effects model. For non-normally distributed variables, data were described as the median (P25, P75) and analyzed using non-parametric tests. A *p*-value of less than 0.05 was considered statistically significant.

## 3. Results

### 3.1. Patient Information

**Figure 3:**
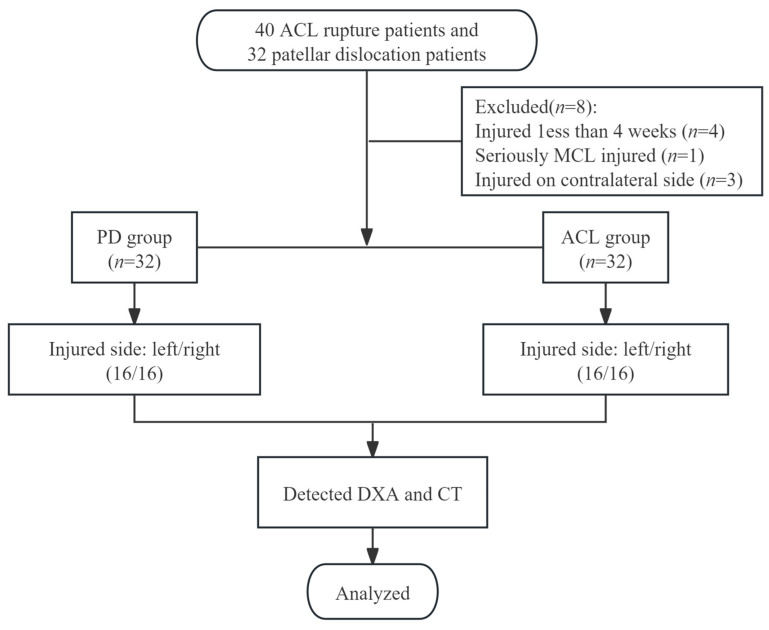
Flow diagram of eligible and included participants. Abbreviations: ACL, ACL rupture group; PD, patellar dislocation group.

From April 2023 to August 2024, a total of 40 ACL rupture patients were screened. Of these, eight participants were excluded: four had injuries lasting less than 4 weeks, one had a severe MCL injury, and three had injuries on the contralateral side. As a result, 32 patients with ACL rupture (ACL group) were eligible for inclusion. An additional 32 participants with patellar dislocation (PD group) were also included in the study (Figure 3).

The time from injury was significantly longer in the PD group compared to the ACL rupture group (*p* < 0.01). No significant differences were observed for other participant characteristics (Table 1).

### 3.2. BMD of DXA

The results of BMD measurements by DXA are shown in Table 2. None of the patients were diagnosed with primary osteoporosis. However, BMD at the MCF, MCT, and LCT in the PD group were significantly lower than in the ACL group (*p* = 0.000, 0.0003, 0.017, all <0.05).

### 3.3. HU Value of CT

The HU values of cortical bone in the femur and tibia differed significantly between the ACL rupture group and the PD group, whereas the HU values of cancellous bone in both the femur and tibia showed no significant difference (Table 3).

For cortical bone, the HU values in the PD group were significantly lower than those in the ACL rupture group at the posterior femur in the SP, medial tibial plateau in the CP, medial condyle and shaft transition in the CP, lateral condyle and shaft transition in the CP, and posterior tibia in the SP (*p* < 0.05). In contrast, at the lateral condyle articular surface and lateral tibial plateau in the CP, the HU values in the ACL rupture group were significantly lower than those in the PD group (*p* < 0.05).

### 3.4. The Relationship Between BMD and HU Value

A linear mixed model analysis was conducted on the BMD and HU values of ACL and PD patients, with the results presented in Table 4. In our model, the fixed effects included HU values measured at different sites, while the random effects accounted for variations among individual patients and different groups, with random intercepts to account for inter-individual and inter-group differences.

The relationship between BMD and CT can be expressed as “BMD_ij_ = β0 + β1 ✖HU_ij_ + (1|Patient Group) + ϵ_ij_”, where β0 is the fixed intercept, β1 is the fixed effect of the HU value, and (1|Patient Group) represents the random intercepts for patient grouping.

The BMD of the MCF was significantly correlated with the HU value of the medial condyle in the CP (*p* < 0.01). The BMD of the LCF showed significant correlations with the HU values of cancellous bone in the SP of the lateral femoral condyle, cortical bone of the lateral condyle articular surface, and both the anterior and posterior femur in the SP (*p* < 0.05).

The BMD of the MCT was significantly correlated with the HU values of cancellous bone in the CP of the medial condyle, cortical bone of the tibial plateau, and posterior tibia in the SP (*p* < 0.05). Additionally, the BMD of the LCF was significantly correlated with the cortical bone HU value of the lateral tibial plateau in the CP (*p* < 0.05).

## 4. Discussion

Our study found that both ACL rupture and PD led to a reduction in knee joint BMD. Surgery is an effective method for addressing joint instability. However, BMD continues to decline postoperatively, which can hinder the healing of the graft-tendon-bone junction. Therefore, understanding the characteristics of post-injury joint BMD in a more convenient and comprehensive way is crucial for guiding surgery and precision rehabilitation. However, there is currently a lack of research on the use of CT for assessing knee joint BMD as well as a lack of studies exploring the changes in knee joint BMD following ACL rupture and PD. This is the first study to explore the BMD changes in patients after ACL rupture and PD using both DXA and CT and to compare these changes between the two groups. The level of evidence in our study is relatively high. The significance of our research lies in the finding that the knee joint BMD and HU values of PD patients were lower than those of ACL rupture patients, with HU values providing a more convenient method for reflecting joint bone content compared to DXA. This information can help guide surgery and precision rehabilitation, ultimately promoting tendon–bone healing after surgery.

The results indicate that the degree of bone loss in the knee joint is greater in PD patients compared to ACL rupture patients. Additionally, we found that bone loss predominantly involved cortical bone, particularly in the tibia. Possible explanations for this finding include the following: First, PD patients generally have a longer duration of injury compared to ACL rupture patients. A longer injury duration can lead to a more prolonged period of limited physical activity, reducing the amount of movement compared to pre-injury levels, which may contribute to the reduction in joint bone mass. This finding aligns with the IKDC score results, which showed that ACL rupture patients had significantly higher IKDC scores than PD patients. Furthermore, all the PD patients included in this study had experienced two or more patellar dislocations, and many of these patients exhibited a significant fear of reinjury. This fear likely further limited the intensity of their physical activity. Recent studies support our findings, indicating a decrease in BMD in the knee joint following ACL rupture and ACL reconstruction (ACLR). A reduction in BMD of the medial femoral condyle was observed within the first 4 weeks post-ACL rupture, with a more pronounced decline in overall knee joint BMD by the 10th week [26]. Additionally, BMD in the injured leg was significantly reduced compare to the uninjured leg following ACLR. Even 26 months after reconstruction, BMD in the affected knee remained 16% lower than preoperative levels [8], and this decline may persist for over 10 years post-ACLR [9]. During reconstruction, the local BMD in the knee decreases, leading to loosening of the bone tunnel, weakening bone strength at the tendon–bone attachment site, and impairing the organization of transitional tissue at the tendon–bone interface. This process enlarges the bone tunnel, which hinders the healing of the reconstructed ligament and tendon–bone junction [27]. Studies have confirmed that in an osteoporotic ovine model, a decrease in BMD following rotator cuff reconstruction surgery can reduce the tendon modulus of the reconstructed ligament [28]. Following PD, patients may experience a fear of movement, which leads to reduced physical activity. Previous studies have shown that exercise can promote the expression of osteogenic factors, both at the gene and protein levels, accelerating osteoblast proliferation and differentiation, while also reducing osteocyte apoptosis, thereby strengthening bone quality [29]. However, current research has not yet focused on the changes in knee joint BMD following PD nor on how to intervene in response to the BMD decline caused by joint instability. Furthermore, studies exploring appropriate interventions and their impacts on BMD after such injuries are lacking. The results of our study suggest that PD patients may require more targeted interventions for knee joint BMD, such as early and precise rehabilitation exercises, which may also promote tendon–bone healing post-surgery.

The results of our study showing the correlation between BMD and HU values indicate a high degree of association between these two measures, with CT providing a more precise, comprehensive, and convenient reflection of knee joint BMD compared to DXA. Unlike DXA, which measures BMD in two dimensions, CT can distinguish between cancellous and cortical bone and perform three-dimensional measurements. This distinction is particularly important, as factors such as osteophyte formation or calcification at the joint margins can affect the accuracy of DXA measurements [30,31]. CT has a higher resolution and sensitivity, allowing for a more comprehensive assessment of BMD [32,33]. Clinically, when a patient is injured, CT not only allows for the assessment of BMD but is also more commonly used to diagnose bone continuity issues [34,35]. With a single scan, multiple concerns can be addressed, making it more convenient. Previous studies have shown that quantitative CT (QCT) measurements of cancellous BMD are closer to the standard value of grayscale BMD and are better and more accurately able to reflect bone metabolism changes compared to DXA when diagnosing osteoporosis [36]. However, research on QCT for knee joints is still limited. This study demonstrates that CT and DXA are highly correlated when assessing knee joint BMD in cases of ACL rupture and PD, with CT outperforming DXA in terms of accuracy, reliability, and comprehensiveness. Furthermore, this study provides a foundation for future research on QCT for knee joints.

In conclusion, this study reveals that both ACL rupture and PD lead to a decrease in knee joint BMD, with a more significant reduction observed in PD patients. Additionally, CT offers a more precise assessment of BMD compared to DXA. These findings not only support surgical decision making but also provide a basis for developing more accurate rehabilitation plans, ultimately promoting tendon–bone healing after surgery.

## 5. Conclusions

PD patients had lower BMD and HU values in the femur and tibia compared to patients with ACL ruptures, particularly in the cortical bone of the tibia. There is a strong correlation between HU value and BMD. CT provides a more precise assessment of BMD changes and may serve as a valuable tool for monitoring joint health and guiding rehabilitation strategies post-injury.

## Figures and Tables

**Table 1:** Participant characteristics.

Characteristics	ACL (*n* = 32) Mean (SD)	PD (*n* = 32) Mean (SD)
Age (years)	29.02 (7.59)	26.46 (4.55)
Male/Female (*n*/*n*)	17/15	12/20
Height (cm)	170.46 (9.37)	169.38 (7.92)
Weight (Kg)	69.99 (11.64)	67.37 (9.59)
BMI (Kg/m^2^)	23.55 (3.13)	22.84 (3.26)
Time since injury (months)	3.89 (1.66) **	12.37 (9.39)
Tegner score	2.78 (0.55)	3.03 (0.65)
Lysholm score	68.67 (12.41)	69.76(13.35)
IKDC score	69.07 (11.00) *	66.59 (9.82)

Abbreviations: ACL, ACL rupture group; PD, patellar dislocation group; IKDC, International Knee Documentation Committee. * Significant difference compared with CON groups *p* < 0.05, ** *p* < 0.01.

**Table 2:** The BMD of the knee (g/cm^2^).

	ACL Mean ± SD (*n* = 32)	PD Mean ± SD (*n* = 32)	*p*
MCF	1.24 ± 0.23 **	0.99 ± 0.16	0.000
LCF	1.56 ± 0.34	1.47 ± 0.29	0.244
MCT	1.03 ± 0.19 **	0.89 ± 0.13	0.003
LCT	1.07 ± 0.23 *	0.96 ± 0.17	0.017

Abbreviations: BMD, bone mineral density; ROIs, regions of interest; ACL, ACL rupture group; PD, patellar dislocation group; MCF, medial condyle of femur; LCF, lateral condyle of femur; MCT, medial condyle of tibia; LCT, lateral condyle of tibia. * Significant difference compared with PD groups *p* < 0.05, ** *p* < 0.01.

**Table 3:** The HU value of knee CT.

			ACL Mean ± SD (*n* = 32)	PD Mean ± SD (*n* = 32)	*p*
Femur	Cancellous bone in the CP	Lateral condyle	192.96 ± 82.32	187.20 ± 59.28	0.749
	Cortical bone in the CP	Medial condyle articular surface	524.34 ± 111.32	494.15 ± 80.84	0.219
		Lateral condyle articular surface	466.94 ± 90.41 *	507.38 ± 63.69	0.043
		Medial condyle and shaft transition	833.58 ± 151.27	823.94 ± 207.40	0.833
		Lateral condyle and shaft transition	979.28 ± 152.22	929.18 ± 198.05	0.261
	Cancellous bone in the SP	Lateral condyle	215.98 ± 72.42	200.15 ± 56.60	0.333
	Cortical bone in the SP	Lateral condyle articular surface	539.45 ± 107.14	560.52 ± 68.39	0.352
		Anterior femur	744.60 ± 132.84	733.23 ± 118.91	0.719
		Posterior femur	987.99 ± 102.74 **	896.95 ± 123.98	0.002
Tibia	Cancellous bone in the CP	Medial condyle	128.85 ± 67.40	137.04 ± 47.98	0.577
	Cortical bone in the CP	Medial tibial plateau	689.86 ± 141.23 *	621.30 ± 91.34	0.024
		Lateral tibial plateau	447.11 ± 102.14 **	529.86 ± 93.88	0.001
		Medial condyle and shaft transition	871.17 ± 113.65 **	745.16 ± 132.55	0.000
		Lateral condyle and shaft transition	758.75 ± 161.59 **	632.65 ± 137.97	0.001
	Cancellous bone in the SP	Medial condyle	116.45 ± 65.24	118.37 ± 50.28	0.896
	Cortical bone in the SP	Tibial plateau	554.13 ± 118.41	568.31 ± 92.34	0.595
		Anterior tibia	627.91 ± 105.42	598.45 ± 106.43	0.270
		Posterior tibia	1030.18 ± 97.70 **	889.99 ± 104.18	0.000

HU, Hounsfield units; ROIs, regions of interest; ACL, ACL rupture group; PD, patellar dislocation group; CP, coronal plane; SP, sagittal plane. * Significant difference compared with PD groups *p* < 0.05, ** *p* < 0.01.

**Table 4:** The relationship between BMD and HU value.

BMD Site	HU Value Site		β ± SD	*p*
MCF	Cortical bone in the CP	Medial condyle articular surface	0.000959 ± 0.000244	<0.001
		Medial condyle and shaft transition	0.0000517 ± 0.000131	0.694
LCF	Cancellous bone in the CP	Lateral condyle	−0.000203 ± 0.000763	0.790
	Cortical bone in the CP	Lateral condyle articular surface	−0.000234 ± 0.000515	0.649
		Lateral condyle and shaft transition	0.000160 ± 0.000198	0.419
	Cancellous bone in the SP	Lateral condyle	0.00293 ± 0.000807	<0.001
	Cortical bone in the SP	Lateral condyle articular surface	0.00107 ± 0.000454	0.018
		Anterior femur	−0.000589 ± 0.000300	0.049
		Posterior femur	0.000599 ± 0.000297	0.043
MCT	Cancellous bone in the CP	Medial condyle	0.00141 ± 0.000452	0.002
	Cortical bone in the CP	Medial tibial plateau	0.000173 ± 0.000105	0.098
		Medial condyle and shaft transition	−0.000109 ± 0.0000913	0.232
	Cancellous bone in the SP	Medial condyle	0.000617 ± 0.000453	0.173
	Cortical bone in the SP	Tibial plateau	0.000273 ± 0.000117	0.020
		Anterior tibia	0.000108 ± 0.000109	0.321
		Posterior tibia	0.000244 ± 0.000119	0.040
LCT	Cortical bone in the CP	Lateral tibial plateau	0.00121 ± 0.000213	0.016
		Lateral condyle and shaft transition	0.0000144 ± 0.000139	0.917

BMD, bone mineral density; HU, Hounsfield units; ACL, ACL rupture group; PD, patellar dislocation group; MCF, medial condyle of femur; LCF, lateral condyle of femur; MCT, medial condyle of tibia; LCT, lateral condyle of tibia; CP, coronal plane; SP, sagittal plane.

## Data Availability

All relevant data are within the paper.

## References

[1] Liu H., Liu J., Wu Y.W., Ma Y.H., Gu S.J., Rui Y.J. (2021). Changes in local bone mineral density can guide the treatment plan for patients with rupture of the anterior cruciate ligament. Ann. Palliat. Med..

[2] Dai R., Wu Y., Jiang Y., Huang H., Meng Q., Shi W., Ren S., Ao Y. (2024). Epidemiology of Lateral Patellar Dislocation Including Bone Bruise Incidence: Five Years of Data from a Trauma Center. Orthop. Surg..

[3] Moses B., Orchard J., Orchard J. (2012). Systematic Review: Annual Incidence of ACL Injury and Surgery in Various Populations. Res. Sports Med..

[4] Jain N.P., Khan N., Fithian D.C. (2011). A Treatment Algorithm for Primary Patellar Dislocations. Sports Health Multidiscip. Approach.

[5] Kejriwal R., Annear P. (2020). Arthroscopic assessment of patella tracking correlates with recurrent patellar instability. Knee Surg. Sports Traumatol. Arthrosc..

[6] Stropnik D., Sajovic M., Kacin A., Pavlič-Založnik S., Drobnič M. (2020). Early clinical and neuromuscular properties in patients with normal or sub-normal subjective knee function after anterior cruciate ligament reconstruction. Arch. Orthop. Trauma Surg..

[7] Forde C., Mortimer C., Haddad M., Hirani S.P., Williams M.A., Keene D.J. (2021). Objectively quantified lower limb strength recovery in people treated surgically or non-surgically after patellar dislocation: A systematic review. Phys. Ther. Sport.

[8] Ejerhed L., Kartus J., Nilsen R., Nilsson U., Kullenberg R., Karlsson J. (2004). The effect of anterior cruciate ligament surgery on bone mineral in the calcaneus: A prospective study with a 2-year follow-up evaluation. Arthroscopy.

[9] Coggan A.R., Coyle E.F. (1987). Reversal of fatigue during prolonged exercise by carbohydrate infusion or ingestion. J. Appl. Physiol..

[10] Inclan P.M., Brophy R.H. (2023). Revision anterior cruciate ligament reconstruction. Bone Jt. J..

[11] Smith T.O., Gaukroger A., Metcalfe A., Hing C.B. (2023). Surgical versus non-surgical interventions for treating patellar dislocation. Cochrane Database Syst. Rev..

[12] Lee K., Al Jumaily K., Lin M., Siminoski K., Ye C. (2022). Dual-energy x-ray absorptiometry scanner mismatch in follow-up bone mineral density testing. Osteoporos. Int..

[13] Deshpande N., Hadi M.S., Lillard J.C., Passias P.G., Linzey J.R., Saadeh Y.S., LaBagnara M., Park P. (2023). Alternatives to DEXA for the assessment of bone density: A systematic review of the literature and future recommendations. J. Neurosurg. Spine.

[14] Hsu J.T., Chen Y.J., Ho J.T., Huang H.L., Wang S.P., Cheng F.C., Wu J., Tsai M.T. (2014). A comparison of micro-CT and dental CT in assessing cortical bone morphology and trabecular bone microarchitecture. PLoS ONE.

[15] Lee H., Park S., Kwack K.-S., Yun J.S. (2023). CT and MR for bone mineral density and trabecular bone score assessment in osteoporosis evaluation. Sci. Rep..

[16] de Bakker C.M.J., Knowles N.K., Walker R.E.A., Manske S.L., Boyd S.K. (2022). Independent changes in bone mineralized and marrow soft tissues following acute knee injury require dual-energy or high-resolution computed tomography for accurate assessment of bone mineral density and stiffness. J. Mech. Behav. Biomed. Mater..

[17] Failla M.J., Logerstedt D.S., Grindem H., Axe M.J., Risberg M.A., Engebretsen L., Huston L.J., Spindler K.P., Snyder-Mackler L. (2016). Does Extended Preoperative Rehabilitation Influence Outcomes 2 Years After ACL Reconstruction? A Comparative Effectiveness Study Between the MOON and Delaware-Oslo ACL Cohorts. Am. J. Sports Med..

[18] Grindem H., Granan L.P., Risberg M.A., Engebretsen L., Snyder-Mackler L., Eitzen I. (2015). How does a combined preoperative and postoperative rehabilitation programme influence the outcome of ACL reconstruction 2 years after surgery? A comparison between patients in the Delaware-Oslo ACL Cohort and the Norwegian National Knee Ligament Registry. Br. J. Sports Med..

[19] Shi H., Ren S., Huang H., Liu H., Liang Z., Yu Y., Li H., Ao Y. (2022). Bilateral Alterations in Isokinetic Strength and Knee Biomechanics During Side-Cutting 1 Year After Unilateral ACL Reconstruction. Am. J. Sports Med..

[20] Dejour H., Walch G., Nove-Josserand L., Guier C. (1994). Factors of patellar instability: An anatomic radiographic study. Knee Surg. Sports Traumatol. Arthrosc..

[21] Tian G., Yang G., Zuo L., Li F., Wang F. (2020). Femoral derotation osteotomy for recurrent patellar dislocation. Arch. Orthop. Trauma Surg..

[22] Ihle C., Maurer J., Ziegler P., Stöckle U., Ateschrang A., Ahrend M.D., Schröter S. (2019). Sporting activity is reduced following medial reefing performed for patellar dislocation. BMC Musculoskelet. Disord..

[23] Blaty T., Krueger D., Illgen R., Squire M., Heiderscheit B., Binkley N., Anderson P. (2019). DXA evaluation of femoral bone mineral density and cortical width in patients with prior total knee arthroplasty. Osteoporos. Int..

[24] Choi K.Y., Lee S.W., In Y., Kim M.S., Kim Y.D., Lee S.Y., Lee J.-W., Koh I.J. (2022). Dual-Energy CT-Based Bone Mineral Density Has Practical Value for Osteoporosis Screening around the Knee. Medicina.

[25] Borchardt G., Nickel B., Andersen L., Hetzel S., Illgen R., Hennessy D., Anderson P.A., Binkley N., Krueger D. (2022). Femur and Tibia BMD Measurement in Elective Total Knee Arthroplasty Candidates. J. Clin. Densitom..

[26] Wohl G.R., Shymkiw R.C., Matyas J.R., Kloiber R., Zernicke R.F. (2001). Periarticular cancellous bone changes following anterior cruciate ligament injury. J. Appl. Physiol..

[27] Lui P.P., Ho G., Shum W.T., Lee Y.W., Ho P.Y., Lo W.N., Lo C.K. (2010). Inferior tendon graft to bone tunnel healing at the tibia compared to that at the femur after anterior cruciate ligament reconstruction. J. Orthop. Sci..

[28] Johnson J.W., von Stade D., Gadomski B., Easley J., Nelson B., Bisazza K., Regan D., Troyer K., Zhou T., McGilvray K. (2022). Modified Alendronate Mitigates Mechanical Degradation of the Rotator Cuff in an Osteoporotic Ovine Model. Am. J. Sports Med..

[29] Zhang S., Huang X., Zhao X., Li B., Cai Y., Liang X., Wan Q. (2022). Effect of exercise on bone mineral density among patients with osteoporosis and osteopenia: A systematic review and network meta-analysis. J. Clin. Nurs..

[30] Watanabe D., Takano H., Kimura T., Yamashita A., Minowa T., Mizushima A. (2020). The relationship of diffuse idiopathic skeletal hyperostosis, visceral fat accumulation, and other age-related diseases with the prevalent vertebral fractures in elderly men with castration-naïve prostate cancer. Aging Male.

[31] Hayden A.C., Binkley N., Krueger D., Bernatz J.T., Kadri A., Anderson P.A. (2022). Effect of degeneration on bone mineral density, trabecular bone score and CT Hounsfield unit measurements in a spine surgery patient population. Osteoporos. Int..

[32] Pickhardt P.J., Pooler B.D., Lauder T., del Rio A.M., Bruce R.J., Binkley N. (2013). Opportunistic screening for osteoporosis using abdominal computed tomography scans obtained for other indications. Ann. Intern. Med..

[33] Berger-Groch J., Thiesen D.M., Ntalos D., Hennes F., Hartel M.J. (2020). Assessment of bone quality at the lumbar and sacral spine using CT scans: A retrospective feasibility study in 50 comparing CT and DXA data. Eur. Spine J..

[34] Michalski A.S., Besler B.A., Burt L.A., Boyd S.K. (2021). Opportunistic CT screening predicts individuals at risk of major osteoporotic fracture. Osteoporos. Int..

[35] Black D.M., Bouxsein M.L., Marshall L.M., Cummings S.R., Lang T.F., Cauley J.A., Ensrud K.E., Nielson C.M., Orwoll E.S. (2008). Proximal femoral structure and the prediction of hip fracture in men: A large prospective study using QCT. J. Bone Miner. Res..

[36] Cheng X., Zhao K., Zha X., Du X., Li Y., Chen S., Wu Y., Li S., Lu Y., Zhang Y. (2021). Opportunistic Screening Using Low-Dose CT and the Prevalence of Osteoporosis in China: A Nationwide, Multicenter Study. J. Bone Miner. Res..

